# How centrioles acquire the ability to reproduce

**DOI:** 10.7554/eLife.25358

**Published:** 2017-03-08

**Authors:** Midori Ohta, Arshad Desai, Karen Oegema

**Affiliations:** 1 Department of Cellular and Molecular Medicine, Ludwig Institute for Cancer Research, University of California, San Diego, San Diego, United States; 1 Department of Cellular and Molecular Medicine, Ludwig Institute for Cancer Research, University of California, San Diego, San Diego, United States; 1 Department of Cellular and Molecular Medicine, Ludwig Institute for Cancer Research, University of California, San Diego, San Diego, United Stateskoegema@ucsd.edu

**Keywords:** centriole, centrosome, cell division, *C. elegans*

## Abstract

A protein called SAS-7 is required for daughter centrioles to become mothers in *C. elegans*.

**Related research article** Sugioka K, Hamill DR, Lowry JB, McNeely ME, Enrick M, Richter AC, Kiebler LE, Priess JR, Bowerman B. 2017. Centriolar SAS-7 acts upstream of SPD-2 to regulate centriole assembly and pericentriolar material formation. *eLife* **6**:e20353. doi: 10.7554/eLife.20353

Centrioles are organelles that have two critical functions. In dividing cells, they recruit a collection of proteins (known as pericentriolar material) to form larger organelles called centrosomes that nucleate microtubules and organize the spindle poles during cell division (Fu et al., 2015; Figure 1A) In non-dividing cells, centrioles are involved in the production of cilia, the tiny hair-like projections that cells use for signaling, sensing and moving extracellular fluid (Drummond, 2012).

**Figure 1.**
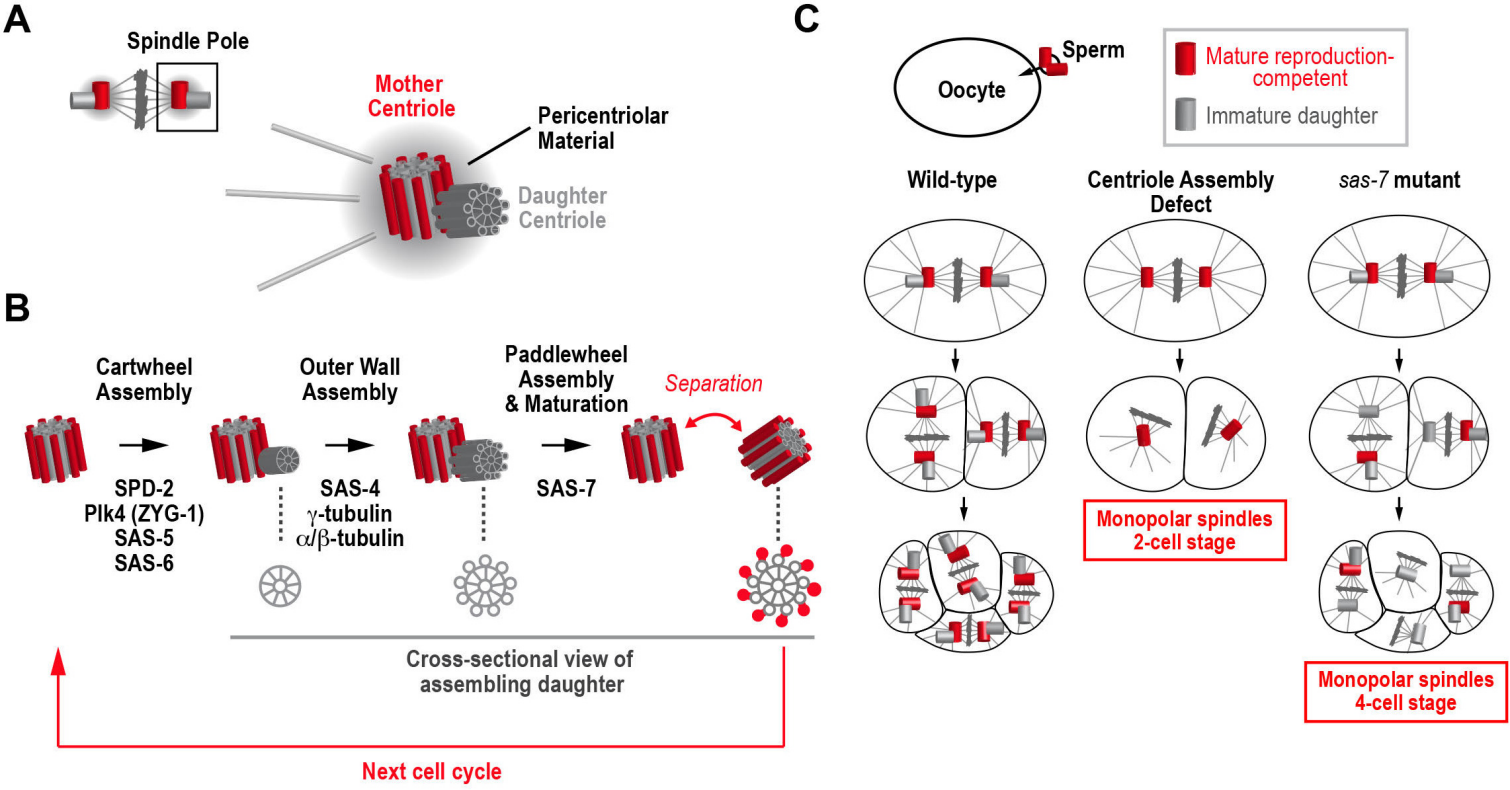
How centrioles duplicate in *C. elegans*. (**A**) Schematic of a metaphase centrosome containing a mature mother centriole that can recruit pericentriolar material and reproduce, and her immature daughter centriole. The pericentriolar material recruited by the mother centriole nucleates microtubules and organizes the pole of the mitotic spindle. (**B**) The steps in the assembly and maturation of the daughter centriole are illustrated, along with the proteins required for each step. Schematics on the bottom show a cross-sectional view of the daughter. Assembly begins when a cartwheel (grey) forms at a right angle to the mother centriole. In the second step, an outer wall made up of nine symmetrically-arranged microtubules (grey) forms around the cartwheel. Assembly of the paddlewheel (a set of protrusions that run along the length of each microtubule; red) and acquisition of the ability to reproduce requires SAS-7. (**C**) The phenotypes observed when a sperm cell containing a wild-type pair of centrioles fertilizes a wild-type egg cell (left column), an egg cell lacking a component essential for daughter centriole formation (middle column), or an egg cell lacking a component required for daughter centrioles to acquire the ability to reproduce (right column).

An individual centriole consists of a central hub called the cartwheel surrounded by an outer wall that contains a nine-fold symmetric array of stabilized microtubules (Figure 1B; Fu et al., 2015; Gönczy, 2012). When a cell is born, it contains two mature centrioles. Concurrent with DNA replication, the centrioles also begin to duplicate, with each centriole giving rise to a new daughter that forms at a right angle to the outer wall of its mother (Figure 1B). By metaphase, the new daughter centriole has a cartwheel and an outer wall. However, while it remains attached to its mother, the daughter centriole is immature because it lacks the ability to recruit its own pericentriolar material and to give rise to its own daughter. As the cell divides into two daughter cells, the new daughter centriole acquires these abilities when it separates from its mother (Figure 1B).

In vertebrates and insects, a pathway for centriole maturation has been identified that requires a specific protein called Cep295/Ana1 (Fu et al., 2016; Izquierdo et al., 2014; Tsuchiya et al., 2016). However, nematodes do not have a Cep295/Ana1 homolog, raising the question of how centrioles mature in these organisms. Now, in eLife, Bruce Bowerman and colleagues – including Kenji Sugioka of the University of Oregon and Danielle Hamill of Ohio Wesleyan University as joint first authors – report the results of experiments on the model nematode *C. elegans* that begin to answer this question (Sugioka et al., 2017). In particular, they have identified a *C. elegans* protein called SAS-7 that is required for centrioles to acquire the ability to reproduce.

The core centriole assembly pathway was discovered in *C. elegans* because the depletion of proteins required for centriole assembly from egg cells leads to a characteristic phenotype (Figure 1C). During fertilization, the sperm cell brings a pair of centrioles into the egg cell, which lacks centrioles. These sperm centrioles duplicate so that the centrosome at each pole of the mitotic spindle contains a mother-daughter centriole pair. After the first round of cell division, each cell of the two-cell embryo inherits two mature centrioles, a mother and a newly mature daughter from the first cell cycle, which both have the ability to reproduce and recruit pericentriolar material to form centrosomes (Figure 1C, left column). In contrast, when a protein required for daughter centriole formation is absent in the egg, the wild-type sperm still brings in a pair of centrioles, but new daughter centrioles fail to form during the first cell cycle, so each cell of the two-cell embryo inherits a single mature centriole, rather than the normal pair of centrioles. Consequently, both cells assemble spindles that have just one pole rather than the normal two (Figure 1C, middle column).

Screens in *C. elegans* identified four proteins whose inhibition leads to monopolar spindles in two-cell stage embryos, indicating that they are essential for the formation of daughter centrioles (Figure 1B): a kinase called Plk4 or ZYG-1 that initiates centriole assembly (O'Connell et al., 2001); SAS-5 and SAS-6, which are required to form the cartwheel (Dammermann et al., 2004; Delattre et al., 2004; Leidel et al., 2005); and SAS-4, which is a structural component of the outer wall of the centriole (Kirkham et al., 2003; Leidel and Gönczy, 2003). A fifth essential component, SPD-2 has two functions: it is required for centrioles to recruit pericentriolar material to form centrosomes and also for daughter centriole formation (Kemp et al., 2004; Pelletier et al., 2004). SPD-2 is the most upstream component in the assembly pathway because it recruits Plk4 kinase to the mother centriole to initiate daughter centriole formation (Delattre et al., 2006; Pelletier et al., 2006). All of these proteins are conserved in vertebrates and are being extensively studied to understand their roles in centriole assembly.

Sugioka et al. study centrioles in *C. elegans* embryos with a mutation in the gene encoding SAS-7. Whereas removing proteins essential for centriole assembly in egg cells leads to monopolar spindles in two-cell embryos, monopolar spindles were not observed until the four-cell stage in *sas-7* mutant embryos fertilized by wild-type sperm. This new phenotype arises because daughter centrioles are able to form during the first cell cycle. The new daughter centrioles separate from their mothers as the first division completes and recruit pericentriolar material to form centrosomes. Thus, both cells of the two-cell embryo have normal bipolar spindles. However, the new centrioles formed in the first cell cycle lack the ability to reproduce and fail to form daughters. Consequently, in the four-cell embryo, the two cells that inherit the sperm centrioles and their daughters assemble normal bipolar spindles, whereas the two cells that inherit the centrioles assembled during the first cell cycle in the embryo have monopolar spindles (Figure 1C, right column). More research is needed to assess whether inhibition of other proteins can cause a similar phenotype (which would indicate that they have a role in daughter centrioles acquiring the ability of to reproduce), indicating a role in acquisition by daughter centrioles of the ability of to reproduce, because the genome-wide RNAi screen that identified the majority of the centriole assembly pathway only monitored one and two-cell embryos (Sönnichsen et al., 2005).

Sugioka et al. also used transmission electron microscopy to visualize centrioles from wild-type and mutant embryos. They found that the wild-type centrioles had a 'paddlewheel' structure that was absent from centrioles assembled in the *sas-7* mutant (Figure 1B). Their results suggest that SAS-7 is required for the formation of this structure.

Sugioka et al. further show that SAS-7 localizes to centrioles and is recruited to them independently of SPD-2. SAS-7 interacts with SPD-2 via a small C-terminal region missing in the mutant protein, and recruitment of SPD-2 to centrioles during interphase, when the daughter centrioles form, is severely compromised in the *sas-7* mutant. Interestingly, assembly of pericentriolar material in mitosis, which also requires SPD-2, is relatively normal, which explains why normal spindles form in two-cell *sas-7* mutant embryos (Figure 1C).

Collectively, the findings of Sugioka et al. indicate that maturation of daughter centrioles involves two events: (1) acquisition of a paddlewheel and the ability to recruit SPD-2 during interphase, which confers on the centriole the ability to reproduce; (2) acquisition of the ability to recruit SPD-2 and assemble pericentriolar material during mitosis to form a centrosome that can organize the spindle pole. SAS-7 is essential for the first event, but not the second, which is why mutations in the gene for SAS-7 affect the competence of centrioles to duplicate, without preventing formation of the spindle pole.

SAS-7 appears to be the functional analog of Cep295 in vertebrates. Like SAS-7, Cep295 recruits the SPD-2 homolog, Cep192, to daughter centrioles during their maturation through a direct interaction with its C-terminus (Tsuchiya et al., 2016). Although Sugioka et al. do not report any sequence homology with Cep295, they do report limited homology between SAS-7, a *Drosophila* protein called Chibby and a human protein called Cby2. Chibby and a paralog of Cby2 (Cby1) are implicated in centriole-to-basal body conversion (Enjolras et al., 2012; Lee et al., 2014), a process that has a central role in the production of cilia. This similarity raises the possibility that the maturation of daughter centrioles and the participation of centrioles in cilia formation may have similar mechanistic underpinnings.
